# Assessing new strategies for TB diagnosis in low- and middle-income countries

**DOI:** 10.1186/1753-6561-8-S4-O12

**Published:** 2014-10-01

**Authors:** Afranio Lineu Kritski, Gisele Huf, Martha Maria Oliveira, S Bertie Squire, Antonio Ruffino-Netto

**Affiliations:** 1 Federal University of Rio de Janeiro, Rio de Janeiro, Brazil; 2 ENSP-Fiocruz, Rio de Janeiro, Brazil; 3 Rede TB, Rio de Janeiro, Brazil; 4 Liverpool School Public of Health, Liverpool, UK; 5 Faculdade de Medicina USP- Ribeirao Preto, São Paulo, Brazil

## 

We will report the current situation of tuberculosis globally and in Brazil, the need for new strategies towards tuberculosis control, focusing on new diagnostic technologies. Critical comments are given on the state of the art regarding the evaluation of new health technologies, degree of scientific evidence needed, evaluation of clinical impact, cost-effectiveness of incorporation into the health system and the social impact.

Currently, the given pragmatic approach indicates that it is not appropriate to conduct an investigation on the incorporation of new technologies only in a "purely experimental" manner in clinical research centers. Research and practice clinical processes become intertwined and the main outcomes to be considered are the patient's health and actions creating a more effective health system in which the new technology will be incorporated. In this new scenario, it is essential that the academic biomedical areas reformulate their undergraduate curricula to include courses that address the development of new technologies, including the assessment of clinical impact, and economic and social incorporation of these new technologies into the current health system that will influence the future practice of their graduate students. In parallel, only through collaborative activities between academics, health service providers (public or private), producers of raw materials, laboratories and representatives of civil society will it be possible to conduct such studies under routine conditions in demonstration areas to enable an analysis appropriate to the relevance of the incorporation of new technologies in the country.
